# Heterogeneity and Actin Cytoskeleton in Osteoclast and Macrophage Multinucleation

**DOI:** 10.3390/ijms21186629

**Published:** 2020-09-10

**Authors:** Jiro Takito, Masanori Nakamura

**Affiliations:** Department of Oral Anatomy and Developmental Biology, School of Dentistry, Showa University, 1-5-8 Hatanodai, Shinagawa, Tokyo 142-8555, Japan; masanaka@dent.showa-u.ac.jp

**Keywords:** actin, fusion, environment-dependent signaling, foreign body giant cells, macrophage, mechanotransduction, podosome, multinucleation, osteoclasts

## Abstract

Osteoclast signatures are determined by two transcriptional programs, the lineage-determining transcription pathway and the receptor activator of nuclear factor kappa-B ligand (RANKL)-dependent differentiation pathways. During differentiation, mononuclear precursors become multinucleated by cell fusion. Recently, live-cell imaging has revealed a high level of heterogeneity in osteoclast multinucleation. This heterogeneity includes the difference in the differentiation states and the mobility of the fusion precursors, as well as the mode of fusion among the fusion precursors with different numbers of nuclei. In particular, fusion partners often form morphologically distinct actin-based linkages that allow two cells to exchange lipids and proteins before membrane fusion. However, the origin of this heterogeneity remains elusive. On the other hand, osteoclast multinucleation is sensitive to the environmental cues. Such cues promote the reorganization of the actin cytoskeleton, especially the formation and transformation of the podosome, an actin-rich punctate adhesion. This review covers the heterogeneity of osteoclast multinucleation at the pre-fusion stage with reference to the environment-dependent signaling pathway responsible for reorganizing the actin cytoskeleton. Furthermore, we compare osteoclast multinucleation with macrophage fusion, which results in multinucleated giant macrophages.

## 1. Introduction

**Figure 1:**
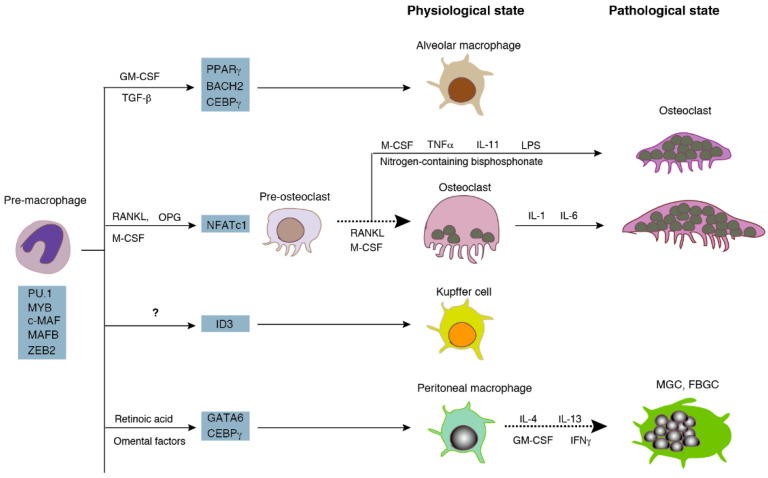
Differentiation of osteoclasts and multinucleated macrophages. Macrophages and osteoclasts are differentiated from the same lineage. Osteoclasts are multinucleated cells at the physiological state. Multinucleated giant macrophages are formed by infection, inflammation, and the foreign-body reaction. The broken arrows indicate the processes discussed in this review. Adopted and modified from [1]. MGC: multinucleated giant cells, FBGC: foreign body giant cells, GM-CSF: granulocyte-macrophage colony-stimulating factor, IFN: interferon, IL; interleukin, LPS: lipopolysaccharide, M-CSF: macrophage colony-stimulating factor, OPG: osteoprotegerin, RANKL: receptor activator of nuclear factor kappa-B ligand, TGF: transforming growth factor, TNF: tumor necrosis factor.

Both osteoclasts and macrophages are differentiated from monocyte/macrophage lineage cells (Figure 1) [1]. Mononucleated precursors become multinucleated osteoclasts via cell fusion during differentiation. Multinucleated osteoclasts live in the bone tissue and resorb bone to maintain the homeostasis of serum calcium and the skeleton at the physiological state. On the other hand, macrophages are distributed in many organs as resident macrophages, such as microglia in the brain, Kupffer cells in the liver, and peritoneal macrophages. Resident macrophages exert their functions, i.e., the phagocytosis of cellular debris and foreign materials, in the form of mononucleated phagocytes [2]. Pathological conditions, such as inflammation and infection, induce the fusion of resident macrophages to form multinucleated giant cells (MGC) [3,4,5]. Furthermore, the persistent presence of foreign micro-organisms or non-phagocytable materials contributes to the formation of the foreign body giant cells (FBGC). Multinucleation reinforces the phagocytic activity of macrophages and osteoclasts. Fused macrophages are able to phagocytose large materials that mononucleated macrophages are unable to degrade [6]. Similarly, large osteoclasts with a high number of nuclei exhibit a higher bone-resorbing activity than small osteoclasts [7,8].

Cell fusion, the merger of independent cells, occurs during the fertilization and organization of multinucleated cells using different mechanisms [9,10,11]. The process of cell fusion can be divided into three stages: pre-fusion, membrane fusion, and post-fusion stages. The pre-fusion stage involves the differentiation, recognition, and adhesion of fusion precursors [9]. The membrane fusion stage includes the deformation and merging of plasma membranes. The reshaping of plasma membranes and the mixing of cytoplasm occurs at the last stage. Osteoclast differentiation is encoded by the lineage-determining transcription program, and the receptor activator of nuclear factor kappa-B ligand (RANKL)-dependent signaling pathway. Many molecules are reported to be involved in osteoclast and macrophage multinucleation, although the cellular mechanism of this fusion remains unknown [4,12]. Recent studies have revealed an unexpected heterogeneity of osteoclast multinucleation, including differences in the differentiation states and nuclearity of precursors, in the speed of fusion, and in the actin-based structure that links fusion partners at the pre-fusion stage. This review highlights the significance of environmental signaling in osteoclast and macrophage multinucleation with the aim of elucidating the origin of these heterogeneities.

## 2. Heterogeneity in Multinucleated Phagocytes In Vivo

### 2.1. Diversity of Osteoclasts In Vivo

#### 2.1.1. Site-Specific-Osteoclasts

As shown in Figure 1, macrophages distribute as a subset of macrophages at distinct sites. The human body contains over 60 different bones with unique structures. Whether osteoclasts can be divided into subsets like macrophages is an extensively discussed hypothesis [13]. The idea of bone site-specific-osteoclasts comes from the observation of bone-site-specific osteopetrosis, a bone disease with an increased bone mass due to the absence of—or dysfunctional—osteoclasts. In osteoprotegerin (a decoy receptor for RANKL) transgenic mice, although there is an increase in the bone mass of the long bone and vertebrae, there is no abnormality in tooth eruption, another index of osteopetrosis [14]. This suggests the difference in osteoclastogenesis between long bones and the jaw. The idea of site-specific-osteoclasts is supported by the difference in the expression of osteoclast-specific genes. Long bone osteoclasts express higher levels of cysteine proteinases than calvarial osteoclasts [15]. Calvarial osteoclasts express 25-fold higher levels of tartrate-resistant acid phosphatase (TRAP) than long bone osteoclasts [16]. Furthermore, osteoclasts in the proximal metaphysis in the long bone express higher levels of TRAP than distal metaphyseal osteoclasts, suggesting site-specific differences in osteoclasts in the same bone [17].

There are two different types of phagocytes that function like osteoclasts in vivo. The first, chondroclasts, are morphologically indistinguishable from osteoclasts, live in the cartilage, and degrade mineralized cartilage [18]. The second, mononucleated perivascular phagocytes, also known as septoclasts, resorb the transverse septum, the uncalcified hypertrophic cartilage of the growth plate [19,20]. Septoclasts produce proteinase cathepsin B and form ruffled border membranes similar to osteoclasts.

The size of osteoclasts, that is the number of nuclei per cell, is another good indicator of the heterogeneity of osteoclasts. An individual osteoclast in the mouse bone has an average number of five nuclei at the physiological state [21]. However, various sizes of osteoclasts have been reported in mice under the different experimental settings. Only osteoclasts with a single nucleus have been found in dendritic cell-specific transmembrane protein (DC-STAMP)-, d2 isoform of vacuolar ATPase (ATP6v0d2)-, and osteoclast stimulatory transmembrane protein (OC-STAMP)-deficient mice, respectively [22,23,24]. On the other hand, giant osteoclasts with more than 10 nuclei have been found in the mouse model of Cherubism [25]. Large osteoclasts are also observed in mice after the administration of nitrogen-containing bisphosphonate [26].

#### 2.1.2. Mode of Osteoclast Multinucleation In Vivo

Although studies on the dynamics of osteoclasts in vivo are scarce, they would provide a principle for the in vitro study of osteoclast fusion. The nuclear kinetics of osteoclasts revealed the lifetime of dog osteoclasts [27]. There are about nine osteoclasts in the evolving secondary Harvesian systems of the dog rib. Each osteoclast has an average number of nine nuclei. The incorporation of tritiated labeled nuclei into the existing osteoclasts was first detected and reached the maximum at 2 days and 4 days after injection, respectively. The transit time of the labeled nucleus is 11.5 days. The number of nuclei per osteoclast is nearly constant during the experiments (15 days). In this study, the authors estimated the lifespan of the osteoclasts to be equal to that of the whole evolving osteon.

Lineage tracing experiments elucidated the origin and lifespan of mouse osteoclasts [21]. Osteoclasts found in the ossification centers at embryonic day (E) 15 are derived from the embryonic erythro-myeloid progenitor (EMP) lineage. Surprisingly, these osteoclasts survive at least 6 months after birth. Time-course parabiosis and EdU (5-ethynyl-2′-deoxyuridine) labeling experiments indicated that hematopoietic stem cell (HSC)-derived blood leukocytes fuse with existing osteoclasts one at a time every 4–8 weeks. An individual osteoclast has an average number of five nuclei, and this number remains nearly constant during 6 months. The turnover of individual nucleus in an osteoclast is about 2 months. An independent group confirmed the EMP-derived origin of osteoclasts and their fusion with HSC-derived cells [28]. The descendants of EMP-derived yolk-sac macrophages pooled in the spleen have been found to migrate through the bloodstream and reach the injury site to form osteoclasts in the bone healing process in 2 month old mice.

The mechanism of the formation of new osteoclasts during bone healing and inflammation may differ from that in the remodeling bone. The formation of osteoclasts has been examined by extracting the upper right row of molars in young rat mandibles [29]. Mononucleated osteoclast precursors appeared near the periosteum at 2 days after the extraction and moved to the bone surface with the concomitant differentiation. The differentiated precursors committed fusion and became osteoclasts at the bone surface. Because the number of osteoclasts significantly increased, the induced osteoclasts appeared to be newly-formed in this healing model.

### 2.2. Heterogeneity of the Multinucleated Macrophages In Vivo

MGCs appear in the tissues of autoimmune, neoplastic, and genetic disorders [5]. The Langhans giant cell (LGC) found in granulomas are small multinucleated cells up to 20 nuclei. The nuclei are peripherally located in a horseshoe shape. Xanthogranulomas contain Touton giant cells that have multiple clustered nuclei surrounded by a foamy cytoplasm. FBGCs are large multinucleated cells with more than 100 nuclei. In an extraskeletal implantation model, the combination of hydroxyapatite with different microstructures and plasma generated FBGCs with distinct levels of marker protein expression [30].

## 3. Multinucleation of Phagocytes In Vitro

Both osteoclasts and multinucleated macrophages are derived from the same origin, that is monocyte/macrophage lineage [1,4]. The lineage produces monocytes, macrophages, dendritic cells, and osteoclasts. Importantly, osteoclast-like cells are induced to differentiate from the differentiated monocytes, macrophages [31], and dendritic cells [32] in vitro under the suitable conditions. Hereafter, we denote multinucleated cells differentiated in vitro as osteoclast-like cells (OCLs) to discriminate them from genuine osteoclasts in bone. Because OCLs are induced from multiple precursors, they do not represent a single and homogeneous population [33]. Researchers prefer bone marrow cells primed with macrophage colony-stimulating factor (M-CSF) (bone marrow macrophage, (BMM)) and the macrophage cell line RAW 264.7 cells for in vitro osteoclastogenesis. On the other hand, multinucleated macrophages are induced from monocytes and macrophages in vitro, and are both a heterogeneous population consisting of multiple subsets [34,35]. Although various types of resident macrophages are generated by the tissue-specific signals (Figure 1), only a few resident macrophages have the capacity to fuse and form MGCs in vitro. In the interleukin-4 (IL-4)-induced fusion assay, thioglycollate-elicited peritoneal macrophages succeed to commit fusion, but not BMMs, resident peritoneal macrophages, or biogel-elicited peritoneal macrophages [36]. This suggests that thioglycollate-elicited peritoneal macrophages have a competency for cell fusion. The nature of fusion competency should be the subject of future research.

### 3.1. Differentiation of Osteoclasts

#### 3.1.1. Osteoclastogenesis via Canonical and Non-Canonical Pathways

Resident phagocytes are produced via distinct transcriptional programs. The programs consist of common lineage-determining transcription factors, such as PU.1, MYB, and c-MAF, as well as niche-dependent transcription factors [1]. The niche-dependent programs require growth factors, such as M-CSF, granulocyte-macrophage colony-stimulating factor (GM-CSF), and IL-34 [37]. Here we outline the niche-dependent program of osteoclasts, i.e., the RANKL-dependent differentiation pathway [38,39,40]. In the presence of M-CSF, osteoclast differentiation begins with the binding of RANKL presented by osteoblasts or osteocytes to RANK expressed on the surface of monocyte-lineage cells. The binding recruits adaptor proteins such as tumor necrosis factor (TNF) receptor-associated protein 6 (TRAF-6) to the cytoplasmic tail of RANK, leading to the activation of transcription factor, nuclear factor-κB (NF-κB). The activated NF-κB and nuclear factor of activated T-cells, cytoplasmic 2 (NFATc2) induce a master transcription factor, NFATc1 at the initial stage of osteoclast differentiation. NFATc1, in turn, transcribes various osteoclast-specific genes, such as TRAP, cathepsin K, and DC-STAMP at the late stage of differentiation. The differentiation signaling induces the expansion of precursors at the early stage of differentiation and the subsequent cell cycle withdrawal [41]. The cell cycle arrested mononucleated precursors commit fusion at the late stage of differentiation. Interestingly, cell fusion can be separated from the induction of osteoclast-specific genes, such as TRAP. In a previous study, fusion incompetent mononucleated cells, such as DC-STAMP-deficient cells generated in vitro showed the same level of expression of osteoclast-specific genes as control OCLs [22]. In in vitro differentiation assays, OCLs are usually defined as the TRAP-positive cells with more than three nuclei. TRAP-positive mononucleated cells are not counted as OCLs. Similarly, TRAP-positive binucleated cells are not regarded as OCLs, because these cells may be produced by cytokinesis.

RANKL-dependent signal transduction, also called the canonical pathway, is a major route of osteoclastogenesis under both physiological and pathological conditions. In vitro experiments have demonstrated that various other factors induce OCLs in the presence and/or absence of RANKL (Figure 1). These factors use distinct intracellular signaling pathways, which are collectively called the non-canonical pathways [42]. They include TNF-α, IL-1, IL-6, IL-11, lipopolysaccharide, and transforming growth factor-β (TGF-β), many of which are secreted in bone-related diseases, such as osteoporosis, rheumatoid arthritis, and periodontal diseases. Although the non-canonical pathways are clinically important, the mechanism of its multinucleation is largely unknown. Accordingly, we describe below the mechanism of the RANKL-dependent multinucleation of OCLs.

#### 3.1.2. Factors That Affect OCL Differentiation

Studies on RANKL-dependent OCL formation are often associated with controversy and concerns about reproducibility. This may be due to the nature of the assay, which is environment-sensitive. First, most experiments on OCL multinucleation cited in this review use a soluble RANKL as an inducer, whereas the OCL formation depends on the cell-cell interaction between OCL progenitors and osteoblasts [43,44]. Second, OCL multinucleation has an optimal seeding density of OCL precursors [45,46]. The dependence on cell density indicates the potential significance of the cell-cell interactions. Third, most OCL differentiation assays have been performed on plastic dishes or glass, as OCL formation on bone is less efficient. However, osteoclast fusion in vivo occurs only in bone. Finally, OCL formation critically depends on the serum used. The difference in serum sometimes causes a reproducibility problem. Furthermore, researchers often add a small amount of additives to the culture media to improve the efficacy of OCL multinucleation, such as non-essential amino acids, L-glutamine, and sodium pyruvate, the effect of which has not been elucidated on the precursors during osteoclastogenesis. Because the factors described above change the number of osteoclasts in the end-point population assay, they may also alter the mode of fusion of OCL precursors. Although our knowledge about the in vivo environment for osteoclast fusion is limited, it is desirable to examine the mechanism of OCL multinucleation at least on bone/dentin slices in future.

#### 3.1.3. Behaviors of Individual Cells in a Mass during OCL Fusion

A canonical differentiation pathway was established from large amounts of genetic and biochemical data. The resulting beautifully arranged signaling pathway indicated that differentiation events proceed in a synchronous manner. However, when you monitor OCL fusion using live-cell imaging, you will face a different world [47,48,49]. In the presence of M-CSF and RANKL, the majority of cells are TRAP-positive mononuclear cells, which sometimes form large aggregates. Mononuclear cells migrate, collide with, and detach from other cells. Very rarely, collided cells fuse in a few minutes. Multinucleated cells extend long processes and form metastable connections with other multinucleated cells. Part of the plasma membranes of large multinucleated cells surrounds another mononucleated cell and engulfs it via a process similar to phagocytosis. Surprisingly, large multinucleated cells wiggle for a time and separate into two cells. The canonical differentiation pathway does not predict these cell behaviors.

### 3.2. Differentiation of the Multinucleated Macrophages In Vitro

Although the mechanism of macrophage multinucleation in vivo is poorly understood, monocytes and macrophages stimulated with growth factors and inflammatory cytokines are known to become multinucleated macrophages in vitro [5]. In vitro multinucleated macrophages show heterogeneity [3]. The stimulation of human monocytes by IL-4 with GM-CSF or IL-3 induces the formation of large multinucleated giant cells with an average number of 285 nuclei. In contrast, human monocytes stimulated with interferon-gamma (IFN-γ) and GM-CSF produce small multinucleated cells with an average of 16 nuclei.

Because a standard protocol for macrophage multinucleation has not yet been established, our understanding of the mechanism of signal transduction leading to macrophage fusion is incomplete. Despite this, the role of DC-STAMP in macrophage fusion is widely recognized. The combination of cytokine IL-4 and IL-13 induces FBGCs from human monocytes [3,50]. IL-4 activates signal transducer and activator of transcription factor (STAT)-6, resulting in the inhibition of STAT-1 [51]. This inhibition in turn induces the expression of OC-STAMP and DC-STAMP, leading to the formation of FBGCs. LGCs are induced from human monocytes co-cultured with autologous T cells in the presence of concanavalin A [52]. In this assay, the role of T cells can be replaced by CD40 ligand and IFN-γ. CD40 ligand presented by T cells binds with CD40 on the monocytes and upregulates the expression of DC-STAMP via NF-κB and MAP kinase signaling. Therefore, the induction of DC-STAMP is critical for both macrophage and OCL multinucleation. However, the transcription of DC-STAMP is regulated by PU.1 and NF-κB during macrophage multinucleation, whereas it is controlled by c-Fos and NFATc1 during the OCL multinucleation [53]. In vitro macrophage multinucleation, like osteoclastogenesis, is sensitive to culture conditions, such as the culture media, seeding cell density, and the properties of the matrix [36,54,55].

## 4. Mode of Phagocyte Fusion

### 4.1. OCL Fusion Occurs in a Heterotypic Manner

**Figure 2:**
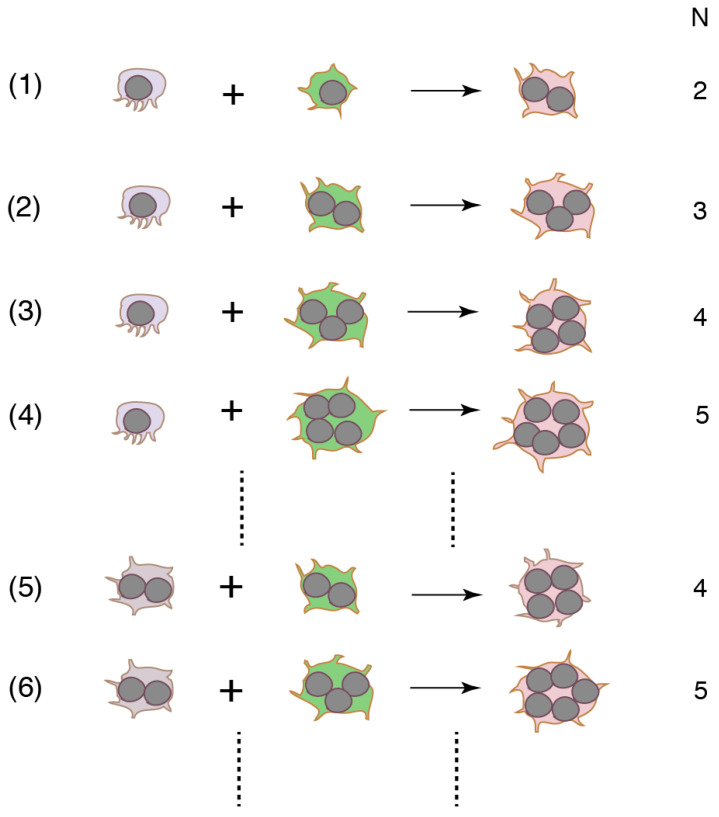
Distinct combinations of precursors form multinucleated cells. Because the fusion precursors are heterotypic, the precursors are coded by different colors. Although the number of nuclei in the multinucleated cells is the same, the cells may be generated by different mechanisms. N: number of nucleus in multinucleated cells after fusion.

Cell fusion with various combinations of precursors with different numbers of nuclei produces various sizes of OCLs (Figure 2). The first fusion must occur between two mononucleated cells. Small OCLs become bigger after rounds of fusion. Because the canonical pathway does not provide the mechanism by which heterogeneity is created, a binucleated cell is expected to be formed via homotypic fusion. However, this was disproved by the experimental results where a RANKL stimulation splits the flow cytometry-sorted DC-STAMP-positive single population into DC-STAMP^high^ and DC-STAMP^low^ populations in mouse BMM and RAW264.7 cells [56]. The DC-STAMP^low^ population showed higher levels of TRAP, OC-STAMP, and CD47 expression than the DC-STAMP^high^ cells. The mixed culture of DC-STAMP^low^ and DC-STAMP^high^ cells yielded a higher fusion rate than the respective homotypic cultures. The results suggest that precursors stimulated by RANKL exhibit heterogeneity in protein expression, and that heterotypic fusion is preferred for OCL multinucleation. Furthermore, different mechanisms appear to be responsible for generating small and large OCLs. Functional blocking experiments show that CD47 promotes the formation of binucleated cells from human monocytes, whereas syncytin-1 stimulates the generation of large OCLs [57,58]. The concept of heterotypic fusion is strengthened by the result that fusion between two mononucleated cells occurs via a small subset of RANKL-stimulated RAW264.7 cells [59]. Newly-formed binucleated cells continue fusion with other mononucleated cells. The secondary fusion proceeds faster than the first fusion. The percentage of the subset is estimated to be only 2.4% of the total RANKL-stimulated cells. Interestingly, the percentage does not change with the incubation time or the concentration of RANKL. These results suggest that a small subset of “founder cells (the multinucleated cell)” fuse with the majority of “follower cells” [59]. Accordingly, the most frequent mode of human monocyte fusion is between mononucleated and multinucleated cells [60].

The heterotypic nature of OCL fusion is manifested by other examples. Mouse wild-type (WT) BMMs are able to fuse with fusion incompetent DC-STAMP-deficient, ATP6v0d2-deficient, and OC-STAMP-deficient BMMs, respectively [22,23,24]. Moreover, OCLs can fuse with non-osteoclastic cells. For instance, OCLs from human monocytes fuse with human myeloma OPM2 cells to generate OCL-myeloma hybrids [61]. RAW264.7 cells primed with RANKL fuse with the mouse melanoma B16F0 cells in the presence of RANKL, TGF-β, and TNF-α [62].

As described above, small and large OCLs are generated via a different mechanism of fusion [58]. OCLs with a different size show the distinct properties. The large OCLs (>10 nuclei) differentiated from RAW 264.7 cells express higher level of integrin αv and β3, cathepsin K, MMP-9, RANK, IL-1 receptor 1 (IL-1 R1), and TNF receptor 1 than small OCLs (<5 nuclei) [63]. In contrast, the expression of a decoy receptor of IL-1, IL-1 R2 and a fusion-related molecule, signal regulatory peptide (SIRPα1) in large OCLs is lower than that of small OCLs. The bone-resorbing activity of large OCLs is preferentially stimulated by IL-1β, probably due to the high expression of IL-1 R1 and low expression of IL-1 R2. These observations are consistent with an idea that large OCLs are more sensitive to the environmental cues than small OCLs. That the different mechanism of fusion results in OCLs with the distinct properties would become an important issue in the pathology of bone diseases.

The above discussion is based on the idea that heterogeneity is inherent in osteoclast differentiation. However, some researchers consider that the heterogeneity may be derived from the non-homogeneity of the precursors. The isolated monocytes and BMMs are inevitably contaminated by different species of cells. The RAW264.7 cell line shows polymorphic properties and changes its phenotypes during passage. To mitigate these drawbacks, several attempts have been made to establish standard conditions for osteoclastogenesis using RAW264.7 cells [64,65,66]. It is interesting to perform fusion experiments in the standard condition in future.

### 4.2. Mode of Macrophage Fusion

To determine whether heterotypic fusion occurs in IL-4-induced macrophage fusion, macrophages primed by IL-4 were mixed with non-treated macrophages. The mixed culture did not produce heterotypic fusion, suggesting that IL-4-induced molecules are needed for both fusion partners [36]. As described above, there are fusogenic and non-fusogenic resident macrophages. In terms of whether cross-fusion can occur between the two populations, fusogenic thioglycollate-elicited peritoneal macrophages can cross-fuse with the non-fusogenic BMMs and biogel-elicited peritoneal macrophages, but not with resident peritoneal macrophages [36]. WT bone marrow cells can fuse with fusion-incompetent DC-STAMP- or OC-STAMP-deficient cells, resulting in the formation of multinucleated macrophages [22,24]. Macrophages spontaneously fuse with colon carcinoma and melanoma cell lines [67]. The idea of heterotypic macrophage fusion is supported by the fact that αMβ2 integrin-overexpressed HEK293 cells are able to fuse with the signal regulatory protein α (SIRPα)-overexpressed HEK293 cells in the presence of IL-4 [68]. Cell fusion does not occur among HEK293 cells, and those overexpressed either αMβ2 integrin or SIRPα alone. Since this experiment used non-fusing HEK293 cells overexpressed with fusion-related molecules, these results should be interpreted carefully. Overall, macrophages can fuse with heterotypic cells. However there are no reports on whether macrophages from a single population generate heterogeneity and commit fusion in a heterotypic manner, as in the OCL precursors.

## 5. Environment-Dependent Signaling in Phagocyte Multinucleation

Heterogeneity is found in many forms in multinucleated phagocytes, from the precursors, fusion, and size, to the function of the multinucleate phagocytes, as described above. To determine what creates these heterogeneities, we will investigate heterogeneity in terms of environment-dependent signaling in this section. Osteoclastogenesis is governed by the lineage-determining and RANKL-dependent pathway. The lineage-determining pathway is a core program shared within the lineage [1], but its role in OCL multinucleation has yet to be elucidated. The RANKL-dependent pathway induces NFATc1 and produces many fusion-related genes, but appears to be irrelevant to the creation of heterogeneity. Although OCL multinucleation is sensitive to environmental cues, such as the properties of the matrix, cell-cell interactions, and growth factors, the role of the environment in osteoclastogenesis has been underestimated. On the other hand, the M-CSF/integrin signaling pathway, an environment-dependent pathway, has been well documented with regards to the reorganization of the actin cytoskeleton. In the following, we verify whether reorganization of actin cytoskeleton via the M-CSF/integrin pathway is involved in the heterogeneity of OCL multinucleation.

### 5.1. Environment-Dependent Signaling in OCL Multinucleation

#### 5.1.1. M-CSF/Integrin Signaling Is Involved in OCL Multinucleation

The reorganization of the actin cytoskeleton is responsible for the mobility, spreading, and bone resorption of osteoclasts. Osteoclasts adhere to the bone via the specialized assembly of actin-rich adhesions, called podosomes. The dynamics and architecture of podosomes is regulated by the M-CSF/integrin signaling pathway, which depends on environmental cues [69,70]. αvβ3 integrin is an important osteoclast integrin distributed in the podosomes [71,72]. M-CSF and integrins are thought to be factors in the proliferation of OCL precursors and the adhesion molecules to the matrix, respectively. Vigorous studies by Teitelbaum’s group found that the two signaling pathways couple in the cytoplasm and form a network that regulates the actin cytoskeleton [73]. Crosstalk of integrin-mediated signaling with other signaling in osteoclastogenesis has been reviewed elsewhere [74].

The perturbation of the M-CSF/integrin signaling causes alterations in the mobility, spreading, and bone resorption of osteoclasts. At the same time, perturbations also change the number and size of OCLs. When isolated rat or rabbit osteoclasts were incubated with M-CSF, the number of large osteoclasts increased by two-fold [75,76], suggesting a novel role of M-CSF as a stimulator of osteoclast fusion. On the other hand, the bone of β3 integrin-deficient mice was found to contain 3.5-fold more osteoclasts than the controls. Conversely, the number of OCLs generated from β3 integrin-deficient BMMs significantly decreased compared to the controls [77]. Retrospectively, the discrepancy may illustrate the environment-sensitive nature of osteoclast multinucleation. The integrin signal is transmitted to the intracellular actin cytoskeleton by the adopter proteins, namely talin and kindlin [78,79]. A linker protein, vinculin, binds with actin and talin and reinforces the actin-integrin linkage [80]. Vinculin-deficient OCLs exhibit retarded multinucleation and a reduced size [81]. Furthermore, kindlin-3 deficient OCLs are smaller than the controls [82]. The signal detected by the integrins is transmitted to the actin filaments. The interference of actin polymerization is expected to alter OCL multinucleation. Indeed, the chemical inhibitors of actin dynamics, namely latrunculin [62,83] and jasplakinolide [84], were found to decrease the number or size of the OCLs. The perturbation of the function of a small GTPase family of proteins, the regulator of actin assembly, such as Wrch1 [85], Rac1 and Cdc42 [86], and Rho [84], was also to affect OCL multinucleation. Therefore, M-CSF/integrin signaling participates in OCL multinucleation.

#### 5.1.2. Interaction between the M-CSF/Integrin Signaling and Canonical Signaling

Because the podosomes appear at the early stage of OCL differentiation [48,69], the M-CSF/integrin-dependent pathway and the canonical pathway co-exist during OCL differentiation. A proto-oncogene, c-Src, is a unique hub in the M-CSF/integrin signaling network [73,84]. OCLs derived from c-Src-deficient spleens [62] or from RAW264.7 cells in the presence of a Src inhibitor [84] are smaller than the controls, suggesting the involvement of c-Src in OCL multinucleation. Interestingly, c-Src binds directly with RANK activated by RANKL and αvβ3 integrin via its SH2 and SH3 domain, respectively [87]. This indicates that c-Src links the M-CSF/integrin-dependent network via the canonical pathway. As such, in regard to whether the canonical signaling contributes to the reorganization of actin cytoskeleton and OCL multinucleation, RANKL promotes the formation of actin ring in matured osteoclasts [88]. RANKL is needed for the multinucleation of OCL precursors [89] and heterotypic fusion between RANKL-stimulated and -nonstimulated RAW264.7 cells [59]. These results suggest that c-Src would link the M-CSF/integrin signaling to the canonical differentiation pathway.

#### 5.1.3. Induction of Gene Transcription by Actin Cytoskeleton Signaling

Gene transcription via actin filaments is best studied in the context of integrin-dependent mechanotransduction [90]. Briefly, the force perceived at the cell surface is transmitted to the cytoskeletons. Cytoskeletons bind with LINC (linker of neucleoskeleton and cytoskeleton) protein complexes, which consist of nesprins localized on the outer nuclear membranes and the SUN (Sad1p, UNC-84) proteins distributed on the inner nuclear membranes. Actin filaments bind with nesprin-1 and nesprin-2. The SUN proteins interact with nuclear lamina proteins lamin-A and -C, nuclear pores, and chromatins. Gene transcription is induced by the activation of YAP (Yes-associated protein) and TAZ ((transcriptional coactivator with PDZ (PSD95/Disc large/Zonula occludens-1)-binding motif)), the nuclear transducers of the Hippo pathways [91,92,93]. Direct force application to the nucleus has been demonstrated to increase the permeability of the nuclear pore, promoting the nuclear translocation of YAP, resulting in gene transcription [94].

During osteoclastogenesis in mouse BMM, RANKL rapidly induces the phosphorylation of YAP1, and downregulates the mRNA and protein levels of YAP1 [95]. The knockdown of YAP1 decreases the induction of osteoclast-specific genes and inhibits OCL multinucleation. The YAP1 and TEAD (transcriptional enhancer associated domain) family of transcription factors complex interacts with AP-1 (activator protein 1) and regulates the induction of NFATc1. On the other hand, the high concentration of extracellular calcium promotes the nuclear localization of YAP/TAZ in OCLs [96]. These results suggest that YAP/TAZ/TEAD signaling induces gene transcription in osteoclastogenesis [97]. The YAP/TAZ activity is regulated by multiple pathways, such as cell-cell adhesion, Wnt signaling, and G-protein coupled receptor (GPCR) signaling [98,99]. In osteoclasts, non-canonical Wnt signaling interacts with the canonical pathway, to reorganize the actin cytoskeleton [100]. As such, gene transcription via the mechanosensitive pathway in OCL multinucleation is a promising area of future research.

#### 5.1.4. The Podosome: A Key to the Heterogeneity?

With regards to how osteoclasts create heterogeneities during differentiation, one possible scenario is that cells perceive the mechanical cues (the composition, rigidity, and topography of the matrix, and shear stress and so on) and translate them into gene expression [90]. The newly expressed genes may change the phenotypes of cells, resulting in the generation of heterogeneity. For osteoclast multinucleation in vivo, the mode of cell fusion may be guided by three dimensional (3-D) cues, such as a collagen/vascular network [101] and interactions with osteoblasts [102] under a physiological state. On the other hand, the cells themselves are a cytoskeleton-based dynamic mechanical entity that interacts with the environment [103]. Podosomes are strong candidates for the generators of heterogeneities in osteoclastogenesis. They are highly expressed in monocytes, macrophages, dendritic cells, and osteoclasts. All these cells show the heterogeneity at the distinct stages of differentiation [33,35]. The podosomes act as the mechanical sensor to the environment and transform their structure and function depending on the developmental program and environmental context [104,105,106]. The transformation of podosomes may proceed without gene expression, because podosomes, self-organized structures, have the properties that allow them to form a large dissipative structure and evolve their structure around the steady state [107]. In this context, podosomes not only have the ability to act as environmental sensors, but also as effectors that create heterogeneity.

### 5.2. Environment-Dependent Signaling in Macrophage Fusion

Studies on implanted biomaterials that cause foreign body-reactions have revealed the significance of the properties of the matrix in the formation of FBGCs in vivo [108]. The significance is clearly shown by experiments using an in vitro FBGC formation model. RAW 264.7 cells and rat BMMs embedded in a collagen-based 3D matrix formed cell clusters and multinucleated macrophages without cytokines [109]. In this model, a change in the compressive modulus, the stiffness of the matrix, caused the differential gene expression of DC-STAMP and CD47, resulting in the diversity of the induced multinucleated macrophages.

#### 5.2.1. Integrins in Macrophage Fusion

Macrophages sense matrix cues and transmit signals to the cytoplasm via integrin receptors. IL-4 with human immunodeficiency virus stimulates β2 integrin-mediated aggregation and MGC formation from human monocytes [110]. Anti-β1 and β2 integrin antibodies inhibit IL-4-induced FBGC formation from human monocytes [111]. Although mouse peritoneal macrophages express αMβ2 and αDβ2 integrins, the former plays a major role in MGC formation induced by IL-4 [112]. Therefore, macrophage and OCL multinucleation depends on αMβ2 and αvβ3 integrins, respectively.

#### 5.2.2. Actin Cytoskeleton and Macrophage Fusion

In macrophages, the dynamic actin-rich structure at the interface between the plasma membrane and cortical actin plays a pivotal role, such as in the form of the phagocytic cup during phagocytosis [2] and as podosomes during matrix degradation [113]. The αMβ2 and αDβ2 integrins are involved in phagocytosis and podosome formation in human phagocytes [114]. The reorganization of the actin cytoskeleton also occurs during macrophage fusion. The chemical inhibitors for F-actin polymerization inhibit IL-13 induced MGC formation from human monocytes [115]. In the IL-4 induced formation of MBGCs from mouse BMM, macrophage fusion is reduced by inhibiting the activation of Rac1 [116]. Arp2/3 and Cdc42 positively regulate IL-4-induced macrophage fusion [114]. Wiskott-Aldrich syndrome protein (WASP), a regulator of the actin cytoskeleton, positively regulates the IL-4-induced macrophage fusion in vitro and the MGC formation in vivo [117]. These results are consistent with the concept that macrophage fusion is regulated by the β2 integrin-dependent signaling via the reorganization of the actin cytoskeleton.

## 6. Multiple Roles of the Actin Cytoskeleton in Phagocyte Multinucleation

The reorganization of the actin cytoskeleton produces diverse actin structures during cell fusion. We will describe the assumed roles of specific actin structures along with the putative temporal order of the fusion events below. Overall, a single OCL fusion consists of a long pre-fusion stage (a few minutes ~1 h) and a short membrane fusion stage (less than a minute) followed by a long post-fusion stage (a few minutes ~1 h) [49,59,62]. Therefore, most of the time of OCL multinucleation is consumed by the pre- and post-fusion events. Readers are recommended to refer to reviews on the general aspects of the structure and function of cytoskeletons [118,119].

### 6.1. Migration of Precursors

Directional migration of fusion precursor cells is a key process for the differentiation and fusion of osteoclasts. Mononuclear precursors in the blood circulation migrate to the bone marrow by external cues such as stromal cell-derived facor-1 (SDF-1 or CXCL12) [120] and sphingosine-1-phosphate (S1P), a lipid mediator [121]. Interestingly, S1P acts as a chemoattractant and chemorepultant via binding to the S1P receptor 1 and S1P receptor 2, respectively [122]. M-CSF and oxysterol, 7α, 25 dihydroxycholesterol regulate the migration of precursors from the bone marrow to the bone surface [123]. This migration appears to be guided by the collagen fiber network that serves as a physical road [101]. The molecular basis of cell migration including chemotaxis depends on the polymerization and depolymerization of actin filaments [124]. The Rho family of GTPases, including Rho, Rac, and Cdc42, reorganize actin filaments and regulate the cell migration in a variety of ways [125]. Hence, defects in the migration of precursors results in a decreased OCL fusion. These molecules include Rac2 [86], adseverin [126], and LIM1 [127]. A force-generating molecule, Myosin X, is known to be also involved in the migration of the precursors [128].

### 6.2. Recognition of Fusion Partners

Migrating cells should recognize other cells and check fusion competency to avoid aberrant fusion. Many molecules are expected to be involved in the recognition process [12], including CD9 [129], CD200 [130], Connexin 43 [131], E-cadherin [132], protocadherin-7 [133], phosphatidylserine [134], and Mac-1/SIRPα [68].

### 6.3. Diversity of the Actin-Based Structure

Live-cell imaging using confocal microscopy has uncovered the variety of actin-based connections between fusion partners at the pre-fusion stage. However, Zambonin Zallone et al. observed similar cell behaviors using an 8 mm movie camera over 30 years ago [135]. They found that chicken monocytes co-cultured with chicken osteoclasts developed contacting filopodia to the osteoclasts and formed protrusions on the surface of osteoclasts before membrane fusion. Thus, recent studies represent a re-discovery and expansion of these initial observations.

#### 6.3.1. Lamellipodia

Lamellipodia along the leading edge of cells is a hallmark of migrating cells. This type of contact is a major form of cell contact, accounting for 68% and 40% of OCL fusion of mouse and human monocytes, respectively (called “podosome-related protrusion” in [62] and “broad contact surface” in [60]) and 64% of the fusion of RAW264.7 cells (called the “leading edge” in [132]). There are numerous thin microvilli-like extensions at the broad contact surface [49,136].

#### 6.3.2. Filopodia

**Figure 3:**
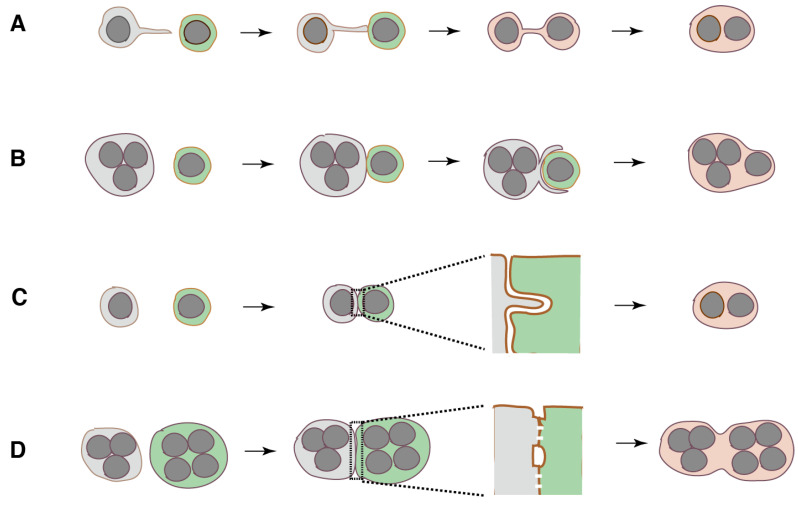
Four types of actin-based linkage between fusion precursors. (**A**), Filopodia-like tube. (**B**), Phagocytic cup-like structure. (**C**), Invasive protrusion. (**D**), Zipper-like structure.

The reorganization of the lamellipodial dendritic actin network results in the formation of protrusions known as filopodia. Filopodium, a narrow cone-shaped protrusion filled with bundled actin filaments, acts as an environmental sensor [137]. A number of reports have suggested that filopodia and filopodia-like protrusions participate in OCL fusion [47,49,62,83,135,138,139,140,141]. Fusion precursor cells use filopodia to identify fusion partners and establish a dynamic connection between two cells before membrane fusion (Figure 3A). A filopodia-like tube that connects two cells called tunneling nanotube (TNT) has been found in many other types of cells. TNTs exhibit diversity in their morphology and in their intercellular communication [142]. At present, there are no clear criteria to differentiate the filopodia-like tubes from TNTs. In RAW264.7 cells, 14% and 18% of OCL fusion occurs via filopodia-like and non-filopodial long protrusions, respectively [62].

Filopodia-like protrusions also participate in the IL4-induced fusion of peritoneal macrophages [117]. This fusion is divided into three types: fusion between mononucleated cells, fusion between mononucleated and multinucleated cells, and fusion between multinucleated cells. An actin-based protrusion connects the fusion precursors in all types of fusion before membrane fusion. The protrusions are statistically classified into short (~3 μm) and long protrusions (~12 μm). Most fusion events are mediated via short protrusion. Fusion via long protrusion proceeds faster than that via the short one. The origin of short protrusion appears to be filopodia at the leading edge.

Interestingly, the fusion partners from RAW264.7 cells transfer membrane phospholipids and GFP (green fluorescent protein)-labeled DC-STAMP from one cell to another via TNTs before membrane fusion [83]. A similar transfer of phospholipid and cell contents during OCL fusion has been independently confirmed by other groups [138,143]. Even nuclei may be transported via the thick connected tube in human monocytes [140]. These results are in accordance with the presence of the content-mixed mononucleated cells in the end-point population assay of OCL formation [138]. During IL-4-induced macrophage fusion, the asymmetric transfer of F-actin from one cell to another occurs before membrane fusion [117]. These observations suggest that filopodia-like tubes act as a mega-scale fusion pore. Further research is needed to determine the “founder”–“follower” relationship between the fusion partners and to identify which cells generate the protrusions. A plausible scenario is that where the tethered partners gives signals to the other to prepare for membrane fusion.

#### 6.3.3. Phagocytic Cup

Because osteoclasts are professional phagocytes specifically adopted to resorb bone [33], it may not be surprising that osteoclasts form the phagocytic cup similar to macrophages. A mononuclear precursor is often trapped by the phagocytic cup-like structure of the fusion partner (Figure 3B). This type of fusion occurs in over 40% of OCL fusion events of human monocytes [60]. It is worth noting that the phagocytic cup may arise from the self-organized actin wave in the podosome field [144,145]. The actin wave has been found to transform into the functional 2D-phagocytic cup in *Dictyostelium* cells [146].

#### 6.3.4. Circumferential Podosomes/Invadopodia Form Invasive Protrusions

At the contact site of fusion partners, one cell forms a protrusion that invades another by deforming the plasma membrane (Figure 3C) [62,138]. Invasive protrusion is derived from the circumferential podosomes/invadopodia. Invadopodia are the podosomes of cancer cells. Protrusions contain actin, PtdIns(3,4)P2, PtdIns(3,4,5)P3, and Tks5 (tyrosine kinase substrate with five SH3 domains), an adaptor protein that plays a central role in podosome/invadopodia formation [62]. Stimulation with RANKL upregulates the expression of Tks5, and the knockdown of its expression inhibits both the formation of protrusions and OCL multinucleation in RAW 264.7 cells. Interestingly, an endocytic protein dynamin is localized in the podosomes [147]. The deletion of the dynamin gene, the knockdown using siRNA, and the inhibition of dynamin activity, cause a decrease in OCL multinucleation [84,138,143]. The fact that F-actin polymerization in podosomes generates the force [148,149] has prompted researchers to postulate that OCL fusion is promoted by the force in the invasive protrusion [62,138,150]. However, this leads to the question of how dynamin participates in the generation of this force.

It is relevant to mention myoblast fusion here, because some researchers have argued that invasive protrusion is a common mechanism for myoblast and OCL fusion [62,138]. During Drosophila embryogenesis, muscle progenitors are specified into muscle founder cells and fusion-competent myoblasts (FCM) [151]. FCMs form the invasive membrane protrusion at the contact site of the founder cells. Because the architecture of the protrusion resembles that of the podosomes, the protrusion is often called the podosome-like structure (PLS). In the PLS, the Arp2/3-dependent branched actin polymerization may generate the force. In the in vitro myoblast fusion using C2C12 cells, myoblast differentiation upregulates the expression of Tks5 and dynamin-2 [152]. Importantly, differentiation induces the isoform switch of Tks5 from the inactive to the active form, which initiates the formation of the PLS. Tks5 interacts with dynamin-2 and actin filaments, forming the super-bundles of actin filaments with a diameter of over 100 nm and an increased stiffness. A dynamin helix recruits 12–16 actin filaments to the outer rim of the helix [153]. The GTP hydrolysis of dynamin stimulates the branched actin polymerization of actin filaments. The cycle of GTP hydrolysis results in the assembly/disassembly of dynamin helix, leading to the dynamic bundling of actin filaments. This mechanism would explain the generation of dynamin-dependent invasive force of the PLS. On the other hand, founder cells form a thin actin carpet underneath the plasma membrane. The asymmetric actin structures at the fusion site produce the invasive and resisting forces between the FCM and founder cells. This balance between the forces has been postulated to bring the two cells into close proximity, thus promoting the engagement of fusogens. Therefore, myoblast fusion shows many similarities with OCL multinucleation, which appears to also operate during macrophage and osteoclast fusion.

#### 6.3.5. Zipper-Like Structure

The OCLs from RAW264.7 cells form a zipper-like structure, an actin-rich superstructure, at their broad contact surface (~160 μm) before membrane fusion (Figure 3D) [48,145]. A similar structure is also found in mouse calvarial osteoclasts [48], OCLs from human blood mononuclear cells [136], and chicken bone marrow cells primed by 1α, 25(OH)_2_D_3_ [154]. The MGCs on the implanted biomaterials in vivo and the IL-4 induced multinucleated macrophages in vitro also form a zipper-like structure [155], although it shows different actin dynamics compared to the counterpart in the OCLs. The zipper-like structure and the podosome belt have a distinct arrangement of podosomal proteins [145]. The force generated by symmetrical actin flow driven by the branched elongation of F-actin in the zipper-like structure is thought to facilitate the adhesion of precursor cells. At the center of the zipper-like structure, the plasma membranes of fusion partners form multiple tight contact sites, in which multiple membrane mergers occur [49]. This observation fits with the model for myotube–myoblast fusion in Drosophila indirect flight muscles [156]. The myoblast and myotube form multiple tight contact sites along a long flattened apposed membranes (~4 μm), which serve as sites for the nascent fusion pore formation, followed by the expansion of pores, leading to cytoplasmic continuity.

### 6.4. Exclusive Zone

The actin cytoskeleton plays various roles in endocytosis [157]. During clathrin-mediated endocytosis, the plasma membrane deforms to form an invagination. The resulting long invagination is pinched off and detached from the membrane, resulting in the formation of a coated vesicle. In yeast endocytosis, the bending site of the plasma membrane is enclosed with a specialized actin meshwork, called the exclusive zone. The exclusive zone expands after the scission of the invagination and prevents the fusion of the newly-formed vesicle with endosomes. Interestingly, the exclusive zone is free of cytoplasmic ribosomes. This property is one of the characteristics of the podosomes of OCLs [48,147]. The restricted area in which the podosome core appears is regarded as an independent cellular entity. The function of the exclusive zone in endocytosis indicates that the podosome-like structure during OCL fusion forms a compartment and isolates the fusion site from the other cellular components. This idea may be supported by the fact that the reorganized architecture of the podosomes, the sealing zone in osteoclasts, compartmentalizes the plasma membrane and delimits the bone-resorbing area. The barrier function of the podosomes at the fusion site can be tested in future studies by investigating the diffusion of membrane proteins.

### 6.5. Fusion Pore Formation

The formation of the fusion pore is the highlight of current models of cell fusion [9,11]. In short, specific cell–cell adhesion molecules, or docking molecules are responsible for keeping two phospholipid bilayers in close contact (<10 nm). A strong bending or protrusion of one or both of the phospholipid bilayers brings them into even closer contact (<2 nm). In this state, the local disruption and rearrangement of the phospholipids occurs between the outer leaflet of the bilayers. Extensive lipid mixing between the outer leaflets results in the formation of the hemifusion stalk. The lateral expansion of the stalk leads to the formation of the hemifusion diaphragm, in which local lipid mixing occurs between the inner leaflets of the bilayers. A resultant merger of the inner leaflets leads to the formation of the fusion pore. The enlargement of the pore following the reshaping of the bilayers completes the membrane fusion.

In OCL fusion, it is speculated that the force generated by the circumferential podosomes promotes the tethering of the bending membranes, resulting in the formation of the hemifusion stalk and the hemifusion diaphragm [62]. Bin/Amphiphysin/Rvs (BAR) domain superfamily proteins generate membrane curvature, in addition to the initiation of actin polymerization at the interface between the membrane and the cortical actin [158]. One of the BAR proteins, insulin receptor tyrosine kinase substrate (IRTKS), is upregulated by RANKL in RAW 264.7 cells, implying its potential involvement in membrane bending [159]. IRTKS interacts with many actin polymerization factors, including dynamin [160]. Therefore, the dynamics of membranes at the fusion sites appear to be under the control of the RANKL signaling. The signaling also changes the composition of the phospholipids in the membranes. It specifically increases the content of phosphatidylethanolamine (PE) during OCL differentiation from mouse BBM [161]. Interestingly, RANKL induces the translocation of PE from the inner to the outer leaflet of the plasma membranes. PE accumulates in the filopodia of fusion precursors. The knockdown of a PE-biosynthetic enzyme in OCL precursors inhibits the translocation of PE and OCL formation.

### 6.6. Professional Fusion Machinery

The above model lacks the central player of membrane fusion, the so-called “fusogen” for the formation of the fusion pore. Candidate molecules include DC-STAMP, OC-STAMP, and syncytins [24,162,163]. Although the lack of each molecule inhibits cell fusion, the mechanism of action remains unknown. However, it is reasonable to assume that some of these molecules form a complex similar to the SNARE complex in the fusion of the transport vesicle with the target organelle [164]. Hereafter, we refer to such a complex, as professional fusion machinery. This machinery should break the energy barrier for membrane fusion and promote pore formation.

### 6.7. Cortical Actin

The current models of osteoclast fusion deal with the fusion of phospholipid bilayers but lack the contribution of the actin cortex [11,62]. The actin cortex is a thin layer (~100 nm thickness) of cross-linked actin filament meshwork underneath the plasma membranes [165]. The interaction between actin filaments and myosin II produces a force, resulting in the lateral tension that contributes to the cell surface tension. Therefore, a decrease in adhesion between the membrane and actin cortex or the local disruption of the actin cortex resulted in the expansion of membranes, as exemplified by bleb formation [166]. The force generated by the actin cortex may counteract the vertical force generated by the accumulation of IRTKS or the circumferential podosomes during the formation of fusion pore.

## 7. Layered Mechanisms Regulate Phagocyte Fusion

**Figure 4:**
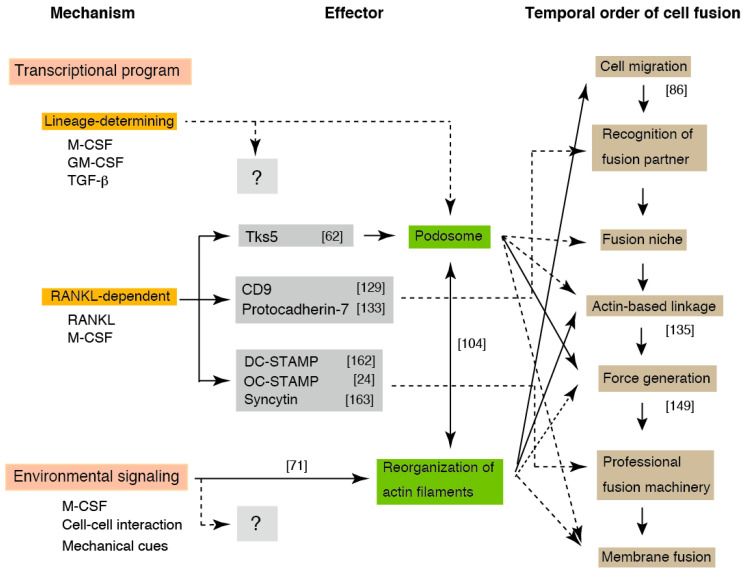
Schematic of the layered mechanism of osteoclast multinucleation. Osteoclast multinucleation is regulated by three interdependent signals. The solid and dashed lines indicate the experimentally demonstrated (specified by the reference number cited in the text) and not demonstrated events, respectively. The fusion-related molecules produced by the lineage-determining program and environmental signaling have not been identified. DC-STAMP: dendritic cell-specific transmembrane protein, OC-STAMP: osteoclast stimulatory transmembrane protein, Tks5: tyrosine kinase substrate with five SH3 domains.

Here, we present a model for osteoclast fusion with reference to the environment-dependent reorganization of actin filaments (Figure 4). Since OCLs can be induced from all monocyte/macrophage lineage cells, several factors may be induced by the lineage-determining transcription factors. We assume that all molecules involved in fusion are prepared via one of three pathways: the lineage-determining, RANKL-dependent, and environment-dependent pathways. The OCL fusion appears to be determined by sequential events consisting of the combined products of the three pathways, rather than a single fusogen. The integration of these three pathways increases the accuracy of fusion and reduces aberrant fusion via the triple-safety mechanism.

The contribution of the environment to osteoclast multinucleation has been underestimated, since β3 integrin, the most important integrin in osteoclasts, is dispensable for osteoclastogenesis [77]. However, the adhesion of precursors to the matrix is essential for the differentiation of OCLs [167], indicating the potential importance of the adhesion signal in osteoclastogenesis. That β3 integrin-deficient BBMs can adhere to the matrix indicates the robustness of the adhesion machinery and the M-CSF/integrin signaling network.

Environmental signaling might elicit many outputs during OCL multinucleation. The recognition of a fusion partner triggers the formation of an exclusive zone at the plasma membrane. Because the exclusive zone is secured from the cytoplasm, the delivery of specific proteins from the cytoplasm to the zone would be an important parameter in OCL fusion [168]. The transported proteins assemble and form the professional fusion machinery, including the fusogen that links partner cells via heterophilic binding. On the other hand, actin-based intercellular linkages, such as the filopodia-like tube or the zipper-like structure, is formed in the zone depending on the environmental cues. The partner cells exchange the cell contents before membrane fusion via the linkage. The local deformation of the phospholipid bilayers would be promoted by the conformational change of the fusogen, in which the force provided by the branched elongation of actin filaments plays a significant role. During this process, the antagonized tension by the actomyosin contraction of cortical actin plays a critical role. With regards to the point at which fusion become irreversible in this model, the establishment of a connection between fusion partners is not an irreversible process, because the partners connected by filopodia-like tubes often breakdown the connection and cancel their pairing. The presence of content-mixed mononucleated cells [138,143] suggests that all of the steps before fusion pore expansion are reversible.

## 8. Perspectives

Heterogeneity is an integral part of osteoclasts throughout their development, from the start of differentiation to the properties of the matured osteoclasts. These heterogeneities may result from the capacity of osteoclasts to adopt their environment. The fusion precursors are able to sense the environment and form distinct fusion-specific actin structures. Podosomes are likely to act as environmental sensors in this process. However, further studies are needed to determine how the M-CSF/integrin signaling network produces distinct actin structures. Macrophage fusion and osteoclast multinucleation show similarities with the induction of DC-STAMP and OC-STAMP, and the formation of the filopodia-like linkage, but dissimilarities with the signaling input responsible for triggering differentiation. Recent studies have suggested functional similarities between macrophages and osteoclasts. Macrophages, like osteoclasts, form the actin-based sealing area and release hydrolytic enzymes into this space to digest materials [169]. FBGCs generated in vivo and in vitro are able to form this sealing zone [170,171]. Conversely, osteoclasts form phagocytic cup-like structures during fusion with a mononucleated cells. Furthermore, the immune function of osteoclasts was recently elucidated [33]. The unexpected versatility and plasticity of phagocytes is likely to be an important topic throughout the next decade.

In the remodeling phase of bone, fusion occurs by the addition of mononucleated cells to existing osteoclasts. Fusion between mononucleated cells is a rare event in vivo, and this process should be the subject of future in vitro research. The mode of fusion in pathological conditions is likely to differ from that in the remodeling phase. Hence, it is critical to reproduce the disease model in vitro and characterize the disease-specific pattern of multinucleation to develop therapeutic strategies against specific bone diseases. The significance of environmental factors in diseases has been shown by a subcellular proteomics study that identified four candidate osteoporosis-related molecules, namely P4HB (β subunit of prolyl 4-hydroxylase), CD36, β1 integrin, and actinin-1 [172]. P4HB catalyzes the hydroxylation of the prolyl residues of preprocollagen. CD36 interacts with collagen type I and type IV. β1 integrin forms a receptor for collagen. Actinin-1 binds with actin filaments. All four molecules are related to matrix (environment)-dependent actin cytoskeleton signaling, described in this review. Over the past three decades, we have devoted our research to elucidating the mechanism of osteoclast differentiation mediated by a genetic program, the RANKL-dependent pathway. It is now time to define the role of the environment in osteoclast differentiation.
